# Effects of menopausal hormone therapy-based on the role of estrogens, progestogens, and their metabolites in proliferation of breast cancer cells

**DOI:** 10.20892/j.issn.2095-3941.2021.0344

**Published:** 2021-11-15

**Authors:** Yu Deng, Hongyan Jin

**Affiliations:** 1 Department of Obstetrics and Gynecology, Peking University First Hospital, Beijing 100034, China

**Keywords:** Menopausal hormone therapy, estrogen, progestogens, breast cancer

## Abstract

Menopausal hormone therapy (MHT) has been widely used for the clinical treatment of symptoms associated with menopause in women. However, the exact nature of the relationship between MHT and the increased risk of breast cancer has not been fully elucidated. The results of the Women’s Health Initiative’s randomized controlled clinical studies showed that estrogen monotherapy was associated with a lower incidence of breast cancer as compared to estrogen-progesterone combined therapy, with an elevated risk of breast cancer. The evidence currently available from randomized trials and observational studies is based on data from different populations, drug formulations, and routes of administration. Even though the risks of MHT and breast cancer have received a great deal of attention, information regarding the unpredictable toxicological risks of estrogen and progestogen metabolism needs to be further analyzed. Furthermore, the diversity and complexity of the metabolic pathways of estrogen and different progestogens as well as the association of the different estrogen and progestogen metabolites with the increased risk of breast cancer need to be adequately studied. Therefore, this review aimed to describe the biological effects of estrogen, progesterone, and their metabolites on the proliferation of breast cancer cells, based on relevant basic research and clinical trials, to improve our understanding of the biological functions of estrogen and progestogen as well as the safety of MHT.

## Introduction

The increasing number of menopausal women in our population has made it necessary to carefully monitor menopausal women’s health care. Menopausal hormone therapy (MHT) is a generic term recommended by The Endocrine Society. It refers to any type of hormone therapy that is administered to menopausal women. This therapy is usually prescribed to alleviate certain menopause-associated symptoms, such as hot flashes, sweating, anxiety, and depression^1–4^. The most frequently used MHTs presently include the estrogen plus progestogen treatment (EPT) and estrogen treatment (ET) based on uterine integrity. Estrogens are classified into natural and synthetic estrogens (**Table 1**). Natural estrogens mainly include estradiol valerate, 17β-estradiol (E2), and conjugated equine estrogen (CEE), whereas synthetic estrogens include nylestriol and ethinylestradiol. Similarly, progestogens comprise natural progestogens and synthetic progestins (**Table 1**). Natural progestogen is represented by progesterone (P4), whereas synthetic progestins mainly include dydrogesterone, medroxyprogesterone acetate (MPA), norethindrone (NET), and drospirenone^6,7^. In addition, estrogen and P4 play critical roles in the development of the mammary gland during puberty and pregnancy^8^. The Women’s Health Initiative (WHI) trial and the Million Women Study (MWS)^9,10^ reported that the use of EPT increased the risk of breast cancer in women; in contrast, results of a French E3N cohort study^11,12^ suggested that estrogen plus P4 did not increase this risk. Therefore, the role of estrogens combined with different progestogens in the development of breast cancer in menopausal women is not determined.

**Table 1:** Classification of estrogens and progestogens

Drug	Classification by structure		Example
Estrogens	Natural estrogen		Estradiol valerate; estradiol (E2)
	Synthetic estrogen		Nylestriol; ethinylestradiol
Progestogens	Natural progestogen		Progesterone (P4)
	Progestins	Retroprogesterone	Dydrogesterone
		17-OH progesterone derivatives (pregnanes)	Medroxyprogesterone acetate (MPA); megestrol acetate; chlormadinone acetate
		17-OH norprogesterone derivatives (norpregnanes)	Gestonorone caproate; nomegestrol acetate
		19-nortestosterone derivatives (estranes)	Norethindrone (NET); norethindrone acetate; lynestrenol; ethinodiol acetate
		19-nortestosterone derivatives (gonanes)	Norgestrel; levonorgestrel; desogestrel; etenogestrel
		19-norprogesterone derivatives (norpregnanes)	Demegestone; promegestone; nesterone; trimegestone
		Spironolactone derivative	Drospirenone

According to reference^5–7^.

Santen et al.^13,14^ used growth kinetic models to show that occult tumors required approximately 16 years and 30 rounds of doubling times before they could be detected by clinical testing; EPT mainly stimulated the growth of pre-existing occult breast cancer and decreased the doubling time from an average of 200 days to 150 days. Hence, estrogen and progesterone may promote the progression of pre-existing occult breast cancer under certain conditions.

Estrogen and progesterone metabolisms are highly diverse and unstable, thereby resulting in unpredictable toxicological risks in women. Previous studies^15–18^ have reported that estrogen metabolites (EMs) and progesterone metabolites may be associated with the risk of breast cancer; however, this aspect has not been adequately investigated. It is therefore essential to review the metabolic pathways of estrogens and different progestogens, to obtain an increased awareness about the role of estrogen and progestogen metabolites in breast cancer.

Because the roles of estrogens and progestogens are highly diverse, yet not clearly known with respect to the progression of breast cancer, our aim was to summarize the epidemiological studies of MHT and breast cancer, the signaling pathways related to E2 and different progestogens, and the pharmacological differences among the various progestogens. This will help to understand the safety of administering MHT as well as its association with the increased risk of developing breast cancer.

## Association of MHT with the risk of breast cancer

The WHI study, which began in 1993, was a long-term, multicenter, randomized, double-blind, and controlled clinical trial that enrolled 27,347 postmenopausal women aged 50–79 years at 40 clinical research centers in the USA. The WHI trial was divided into 2 categories; the WHI EPT Trial and the WHI ET Trial, based on the presence or absence of a uterus, respectively. In the WHI EPT Trial^9^, 16,608 postmenopausal women who had not undergone hysterectomy were randomly selected to receive a daily dose of placebo or a combination of 0.625 mg CEE/2.5 mg MPA. In the WHI ET Trial^19^, 10,739 postmenopausal women who had undergone hysterectomy were randomly selected to receive a daily dose of placebo or 0.625 mg CEE. Compared with women who received the placebo, women who received EPT (CEE + MPA) had a significantly increased risk of breast cancer [hazard ratio (HR): 1.26; 95% confidence interval (CI): 1.00–1.59]^9^. In contrast, women who were administered ET (CEE alone) did not exhibit an increased risk of breast cancer (HR: 0.77; 95% CI: 0.59–1.01)^19^ when compared with women who received the placebo.

The WHI study raised concerns about the association of MHT with the risk of breast cancer. The most used drugs in MHT are CEE, E2, P4, and MPA; however, the most troubling question is their relationships with increased risks of breast cancer. From the data collected in the clinical trials (**Table 2**), it could be inferred that ET, which was only indicated for women who had undergone hysterectomies, had a lower risk of stimulating breast cancer development than EPT (E2 + P4 or E2 + MPA). Moreover, administration of P4 and dydrogesterone appeared to result in a lower risk of inducing breast cancer development than the use of synthetic progestogen, such as MPA^28^. However, a better understanding of the risks of MHT requires a combined knowledge of the molecular mechanisms as well as the biological functions of estrogens and progestogens.

**Table 2:** Results of the clinical studies of menopausal hormone therapy and the risks of breast cancer

Study	Year	Design	Population, *n*	Mean age (range)	Medications	Follow-up years (mean, range)	HR (95% CI)	RR (95% CI)	OR (95% CI)
Rossouw et al.^9^, WHI^†^	2002	RCT	16,608	63.2 (50–79)	EPT (CEE 0.625 mg + MPA 2.5 mg)	5.2 (3.5–8.5)	1.26 (1.00–1.59)	NA	NA
Anderson et al.^19^, WHI	2004	RCT	10,739	63.6 (50–79)	ET (CEE 0.625 mg)	6.8 (5.7–10.7)	0.77 (0.59–1.01)	NA	NA
LaCroix et al.^20^, WHI	2011	RCT	10,739	63.6 (50–79)	ET (CEE 0.625 mg)	10.7	0.77 (0.62–0.95)	NA	NA
Manson et al.^21^, WHI	2013	RCT	10,739 16,608	63.6 (50–79) 63.2 (50–79)	ET (CEE 0.625 mg) EPT (CEE 0.625 mg + MPA 2.5 mg)	13.0 (IQR, 9.1–14.1) 13.2 (IQR, 10.5–14.2)	0.79 (0.65–0.97) 1.28 (1.11–1.48)	NA	NA
Manson et al.^22^, WHI	2017	RCT	10,739 16,608	63.6 (50–79) 63.2 (50–79)	ET (CEE 0.625 mg) EPT (CEE 0.625 mg + MPA 2.5 mg)	18	0.55 (0.33–0.92) 1.44 (0.97–2.15)	NA	NA
Schierbeck et al.^23^, DOPS^†^	2012	RCT	1,006	49.5 (45–58)	ET (E2 2 mg)/EPT (E2 2 mg + norethisterone acetate 1 mg)	10 16	0.58 (0.27–1.27) 0.90 (0.52–1.57)	NA	NA
Beral et al.^10^, MWS^†^	2003	OCS	1,084,110	55.9 (50–64)	ET EPT Tibolone	2.6	NA	1.30 (1.21–1.40) 2.00 (1.88–2.12) 1.45 (1.25–1.68)	NA
Fournier et al.^11^, EPIC-E3N^†^	2005	OCS	54,548	52.8 (40–66.1)	ET EPT (estrogen + progesterone) EPT (estrogen + synthetic progestins)	5.8 (0.1–10.6)	NA	1.1 (0.8–1.6) 0.9 (0.7–1.2) 1.4 (1.2–1.7)	NA
Fournier et al.^12^, E3N^†^	2008	OCS	80,377	53.1 (40–66.1)	ET EPT (estrogen+ progesterone) EPT (estrogen+ dydrogesterone) EPT (estrogen+ other progestogens)	8.1(2–12)	NA	1.29 (1.02–1.65) 1.00 (0.83–1.22) 1.16 (0.94–1.43) 1.69 (1.50–1.91)	NA
Bakken et al.^24^, EPIC^†^	2011	OCS	133,744	58.1 (52.1–61.5)	ET EPT	8.6	NA	1.42 (1.23–1.64) 1.77 (1.40–2.24)	NA
Fournier et al.^25^, EPIC-E3N	2014	OCS	78,353	50.2	ET Current use Past use EPT(Estrogen + progesterone/dydrogesterone) Current use Past use EPT (Estrogen + other progestogen^†^) Current use Past use	11.2	1.17 (0.99–1.38) 1.06 (0.95–1.19) 1.22 (1.11–1.35) 0.96 (0.87–1.06) 1.87 (1.71–2.04) 1.12 (1.02–1.23)	NA	NA
Holm et al.^26^, Diet, Cancer and Health Cohort	2019	OCS	29,243	56 (50–64)	ET EPT, sequential regimens EPT, continuous regimens	17	1.37 (0.95–1.98) 1.27 (0.88–1.83) 1.56 (1.05–2.31)	NA	NA
Vinogradova et al.^27^	2020	OCS	556,109	About 63.3(50–79)	ET Recent users (< 5 years) with longterm use (≥ 5 years) EPT Recent users (< 5 years) with longterm use (≥ 5 years)	20	NA	NA	1.06 (1.03–1.10) 1.15 (1.09–1.21) 1.26 (1.24–1.29) 1.79 (1.73–1.85)

For convenience, we denoted estrogen treatment as ET and estrogen plus progestogens treatment as EPT. RCT, randomized controlled trial; OCS, observational cohort study; WHI, The Women’s Health Initiative; MWS, Million Women Study; EPIC, European Prospective Investigation into Cancer and Nutrition; E3N, Etude Epidémiologique de femmes de la Mutuelle Générale de l’Education Nationale; DOPS, Danish Osteoporosis Prevention Study; Other progestogen^**†**^: chlormadinone acetate, demegestone, dienogest, drospirenone, ethynodiol acetate, gestodene, levonorgestrel, lynestrenol, medrogestone, medroxyprogesterone acetate, megestrol acetate, nomegestrol acetate, norethisterone acetate, and promegestone. HR, Hazard ratio; RR, relative risk; OR, odds ratio.

## Effect of E2 on breast cancer proliferation

### E2 signaling pathways in breast cancer cells

The major endogenous estrogens are E2, estrone (E1), and estriol (E3), among which E2 is the predominant estrogen used by women prior to menopause^29^. Moreover, E1 and E2 are interchangeable through the action of 17β-hydroxysteroid dehydrogenases 1 and 2 (17β-HSD1 and 2)^30^. Estrogen plays a wide range of biological roles in mammary glands, uterine tissues, cardiovascular, musculoskeletal, immune, and central nervous systems through the estrogen receptor (ER)^31^. The ERs are categorized into 2 types, namely ERα and Erβ; ERα is the main receptor for estrogen action in the mammary glands^32^. Estrogens have been shown to stimulate the proliferation of breast cancer cells through genomic and non-genomic pathways. In the genomic pathway, estrogens and their receptors bind directly to the estrogen response elements (EREs) present in the nucleus of breast cancer cells and recruit cofactors to form transcription initiation complexes; these complexes then activate the transcriptions and expressions of proliferation-related target genes, where time is specified in hours or is even delayed for days^33–35^. Non-genomic effects have a faster onset (from seconds to a few minutes) and might be related to interactions with structures in the plasma membrane, with the effects frequently associated with the activation of various protein-kinase cascades^36^. In these pathways^33–35^, E2 can induce rapid cellular effects by binding to the estrogen membrane receptor (mER) or G protein-coupled estrogen receptor (GPER) localized at the plasma membrane, which is required for rapid downstream signaling in the phosphoinositide 3-kinase (PI3K)/Akt kinase pathway as well as the mitogen-activated protein kinase (MAPK) pathway^37–39^.

**Figure 1:**
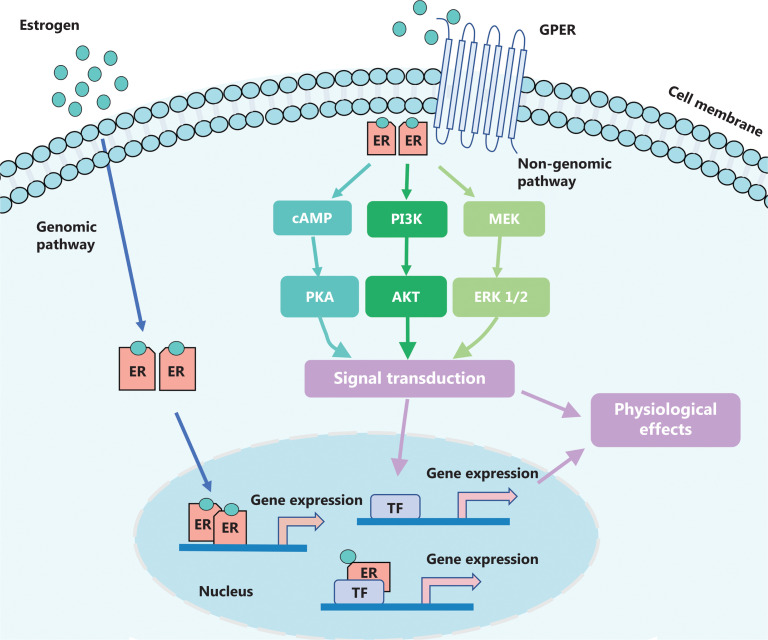
Signaling pathways of estrogen-mediated breast cancer cell proliferation. Estrogen treatment leads to breast cancer growth through genomic and non-genomic pathways. In the genomic pathway, estrogen and its receptors bind directly to specific DNA sequences called estrogen response elements (EREs) and activate gene expressions; additionally, in non-genomic pathways, estrogen signals can occur by plasma membrane localization of ER, or by GPER, to induce rapid cellular effects by activating several kinase cascades, such as cAMP-PKA, PI3K/AKT, and MEK/ERK 1/2. ER, estrogen receptor; GPER, G protein-coupled estrogen receptor; TF, transcription factor. Adapted from Wilkenfeld et al.^33^.

The main non-genomic signaling pathways of estrogens through mER are as follows (**Figure 1**). With respect to the MAPK/ERK pathway, Raffo et al.^40^ reported that the MAPK/ERK pathway was activated within 10 min of administering 10 nM E2 in 2 breast cancer cell lines, namely MCF-7 and LM05-E cells (ER+, PR+). Second, with respect to the cyclic adenosine monophosphate (cAMP)/protein kinase A (PKA) pathway, Aronica et al.^41^ reported that very low estradiol concentrations (a half-maximal dose of 10 pM) caused an increase in intracellular cAMP by increasing membrane adenylate cyclase activity, thereby activating genes that contained the cAMP response element. Third, with respect to the PI3K/AkT pathway, Garrido et al.^42^ reported that within 5, 15, and 25 min of treating MCF-7 cells with 10 nM E2, the PI3K/AkT signaling pathway was activated, thereby stimulating glucose uptake by cells.

The GPER (also known as GPR30) is a seven-transmembrane G-protein-coupled receptor (GPCR) embedded into cellular membranes^43^. Recently published research^44^ highlighted that E2-induced GPER expression promoted proliferation, invasion, and migration of MCF-7 breast cancer cells through the miR-124/CD151 pathway. Filardo et al.^45^ found that stimulation of SkBr3 cells that expressed neither ERα nor ERβ, in the presence of 1 nM 17β-estradiol for 5 min, induced a 6-fold increase of Erk phosphorylation. Moreover, Vivacqua et al.^46^ reported that E2 transactivated the early growth response-1 (Egr-1) promoter sequence and induced Egr-1 expression through the GPER/ERK pathway in SkBr3 breast cancer cells. Additionally, E2 might play an important role in the *in situ* transition of ductal carcinoma in the breast by the GPER signaling pathway^47^. Deng et al.^47^ reported that E2 induced basement membrane disruption in breast glandular ducts by promoting matrix metalloproteinase 3 and interleukin-1β secretion through the GPER/cAMP/PKA and GPER/PI3K/AkT pathways.

### Effects of EMs on breast cancer cells

**Figure 2:**
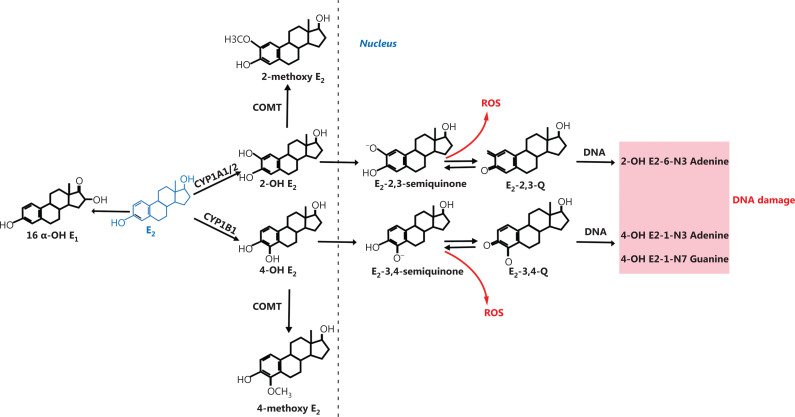
Metabolic pathways of E2. There are 3 pathways of E2 metabolism: the 2-hydroxylation pathway, the 4-hydroxylation pathway, and the 16-hydroxylation pathway, of which the 2-hydroxylation pathway is the main metabolic pathway for E2. 2-OH E2, 2-hydroxyestradiol; COMT, catechol-O-methyltransferase; 4-OH E2, 4-hydroxyestradiol; 16α-OH E1, 16α-hydroxyestrone; E2-2,3-Q, E2-2,3-quinone; E2-3,4-Q, E2-3,4-quinone; ROS, reactive oxygen species.

In addition to ER-induced proliferative effects of the breast cancer cells, E2 is associated with the increased risk of breast cancer due to the DNA toxicity of its metabolites^48^. The metabolism of E2 can be divided into phase I and phase II reactions. In phase I reactions, E2 is metabolized to hydroxyl compounds by the cytochrome P450 enzyme (cytochrome P450, CYP) and in the phase II reaction, the hydroxyl compounds are converted to nontoxic, water-soluble compounds by catechol-O-methyltransferase (COMT). In detail, there are 3 main metabolic pathways for E2 in the liver, namely the 2-hydroxylation pathway, the 4-hydroxylation pathway, and the 16-hydroxylation pathway, of which the 2-hydroxylation pathway is the main metabolic pathway for E2^49^. The metabolic pathways of E2 discussed below are shown in **Figure 2**.

In the 2-hydroxylation pathway, E2 is metabolized to 2-hydroxyestradiol (2-OH E2) by cytochrome P450 enzymes (CYP1A1/2) in the phase I reaction. A previous study reported that 2-hydroxyestrone (2-OH E1) and 2-OH E2 significantly inhibited the hormone-induced proliferation of the human breast cancer cell lines, MCF-7 and T47D^50^. Subsequent *in vivo* tumorigenesis studies showed that tumor growth increases of nude mice treated with 1.5 mg E2 was 335.4%, and that there was no significant increase in the tumorigenesis of the 2-OH E1-treated group, when compared with that of the control group^51^. Moreover, 2-OH E2 is metabolized in the nucleus to E2-2,3-quinone (E2-2,3-Q) by peroxidase, with concomitant production of reactive oxygen species (ROS). The E2-2,3-Q readily binds to DNA to form DNA adducts such as 2-OH E2-6-N3 adenine that can cause DNA damage. In the phase II reaction, 2-OH E2 is further metabolized by COMT to 2-methoxyestradiol, which prevents ROS production and DNA adducts, thereby protecting the cells from the genotoxicity and cytotoxicity of catechol estrogens^52^.

In the 4-hydroxylation pathway, E2 is transformed into 4-hydroxyestradiol (4-OH E2) by the cytochrome P450 enzyme CYP1B1 in the phase I reaction. Lareef et al.^53^ reported that 4-OH E2 increased cell proliferation and induced transformation in MCF-10F cells. Moreover, 4-OH-E2 was reported to induce malignant transformation of breast cells as well as tumor formation in nude mice^18^. The 4-OH E2 might be oxidized to E2-3,4-quinone (E2-3,4-Q) in the nucleus, along with the production of ROS, which increases DNA instability^54^. The E2-3,4-Q easily forms adducts with nuclear DNA, such as 4-OH E2-1-N3 adenine and 4-OH E2-1-N7 guanine, both of which have carcinogenic potential and might lead to DNA damage^55^. In the phase II reaction, 4-OH E2 can be further converted to 4-methoxyestradiol (4-methoxy E2) by COMT, thereby preventing the generation of large amounts of DNA adducts. Zahid et al.^56^ reported that MCF-10F cells oxidized 4-OH E2 to E2-3,4-Q. Additionally, the levels of 4-OH E2-1-N3 adenine and 4-OH E2-1-N7 guanine exhibited a 3- to 4-fold increase when methoxylation of 4-OH E2 was blocked with the COMT inhibitor, Ro41-0960.

In the 16-hydroxylation pathway, E1 and E2 are exchanged through the action of 17β-HSD1 and 17β-HSD2, followed by the transformation of E1 into 2-hydroxyestradiol 16α-hydroxyestrone (16α-OH E1) by the cytochrome P450 enzyme (CYP3A4)^57,58^. The 16α-OH E1 has a stronger estrogenic activity than estradiol, and several studies have reported that 16α-OH E1 significantly increased the expressions of cyclin D1 and cyclin-dependent kinase 2, and promoted cell proliferation in MCF-7 cells^51,59,60^.

There is currently a growing awareness regarding the impact of EMs on menopausal women. There is clinical evidence^15^ suggesting that certain EMs might be risk factors for breast cancer. The Breast and Bone Follow-up to the Fracture Intervention Trial (B˜FIT) cohort^15^ (*n* = 13,784) assessed the relationship between EMs and the risk of breast cancer in postmenopausal women during 12 follow-up years. They found an increased occurrence of the 2-hydroxylation metabolic pathway (HRQ5vsQ1 = 0.69; 95% CI: 0.46–1.05; *P* = 0.01) and also found that a higher ratio of 2/16-hydroxylation pathways (HRQ5vsQ1 = 0.60; 95% CI: 0.40–0.90; *P* = 0.002) were associated with a lower risk of breast cancer. Moreover, previous studies^16,61^ have provided in-depth analyses of the effects of MHT on EMs in postmenopausal women. For example, in a prospective case control study in the Women’s Health Initiative Hormone Trials, the concentration of 2-OH E1 increased 4-fold in the blood, and the ratio of 2-OHE1/16α-OHE1 improved approximately from 0.3 to 1.0, after 1 year of treatment with ET and EPT (*n* = 1,259)^16^. Additionally, Falk et al.^61^ reported that there might be some differences between the estrogen metabolic pathways of women receiving ET and EPT in the WHI Observational Study (*n* = 1,864). In comparison with the effects of EPT, ET was more likely to induce the 2-hydroxylation metabolism pathway than the 16-hydroxylation metabolism pathway. In the future, larger studies are necessary to better determine the relationships between the risks of breast cancer and EM levels with respect to the use of ET and EPT.

In summary, estrogen as well as various EMs regulate genes that are involved in breast cancer cell proliferation. Although the effects of EMs in postmenopausal women have been reported, the underlying mechanisms need further study.

## Effect of progestogens on breast cancer proliferation

**Figure 3:**
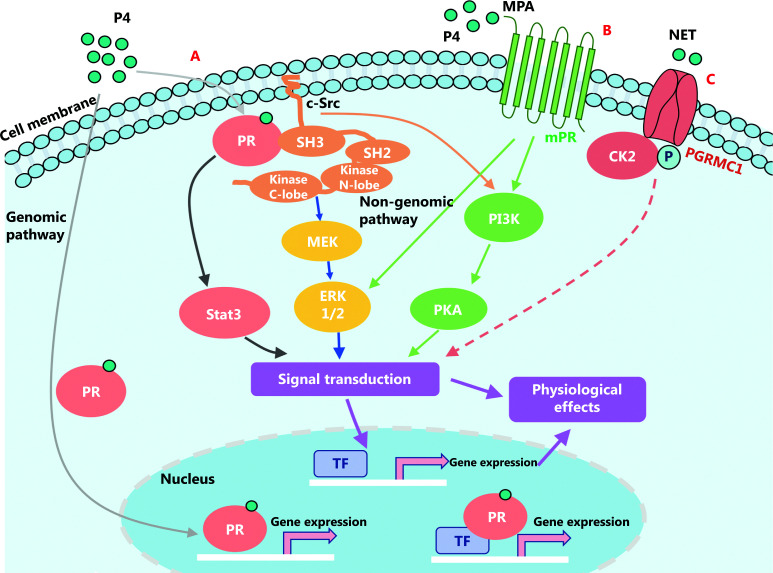
Signaling pathways of progestogens leading to breast cancer growth by genomic and non-genomic pathways. In the genomic pathway, progestogens and progesterone receptors (PRs) bind directly to progesterone response elements (PREs) or other DNA-binding transcription factors to promote gene expressions. Rapid progestogenic effects can be induced by non-genomic pathways, in which progestogens (progesterone, medroxyprogesterone acetate, and norethindrone) through some receptors, such as PRs (A), progesterone membrane receptors (B), and progesterone receptor membrane component 1 (C), activate secondary messenger cascades and regulate gene transcription.

Progestogens exert their effects on their target tissues primarily through genomic as well as non-genomic pathways (**Figure 3**). In the genomic pathway, the progesterone receptor (PR) directly binds to progesterone response elements (PREs) or other DNA-binding transcription factors to modify target gene expressions. In contrast, in non-genomic pathways, progestogens activate secondary messenger cascades through specific receptors, such as PRs, progesterone membrane receptors (mPRs), and progesterone receptor membrane component 1 (PGRMC1), to indirectly regulate gene transcription^62^.

The PRs belong to the steroid hormone receptor family, and the functional region of the PR consists of the DNA binding domain, the ligand-binding domain, and the transcription-activating functions^63^. There are 2 types of PRs, namely PR-A (94 kDa) and PR-B (120 kDa). These 2 types of receptors have different physiological functions; for example, PR-B is a more potent transcription factor than PR-A, but PR-A has an inhibitory effect on the transcriptional activity of PR-B, as well as other receptors, such as estrogen, androgen, and glucocorticoid receptors^64,65^. Under normal physiological conditions, PR-A and PR-B are similarly expressed in breast cells, but breast cancer cells overexpress PR-A more frequently than PR-B^66^. Some studies^67,68^ have suggested that imbalances in the expressions of PR-A and PR-B might play an important role in the early development of breast cancer. Breast cancer patients overexpressing PR-A have worse prognoses and lower chances of disease-free survival than breast cancer patients with PR-B overexpression. Mote et al.^69^ analyzed PR expression in tissue samples of 39 breast cancer patients and found that PR-A > PR-B accounted for 39% (15 out of 39 patients) cases, PR-A = PR-B accounted for 51% (20 out of 39 patients) cases, and PR-A < PR-B accounted for only 10% (4 out of 39 patients) cases. Moreover, Rojas et al.^68^ reported that among 222 PR+ breast cancer tissue samples, 52.3% (116 cases) had PRA-H (PR-A/PR-B) ≥ 1.2.

The mPRs and PGRMC1 lack classical PRs, and their biological functions are not fully understood. The mPRs are novel 7-transmembrane receptors localized on the cell surface, and belong to the progestin and adipoQ receptor (PAQR) family. There are 5 types of mPRS, namely mPRα (PAQR7), mPRβ (PAQR8), mPRγ (PAQR5), mPRδ (PAQR6), and mPRɛ(PAQR9)^70^. The mPRs have similar GPCR functions, and exhibit a rapid response to non-genomic signaling^71^. The membrane-associated progesterone receptor (MAPR) family includes PGRMC1, PGRMC2, neudesin, and neuferricin, all of which contain a cytochrome b5-like heme/steroid binding domain^72^. Zhang et al.^73^ reported a 67.89% positive expression of PGRMC1 in breast cancer patients (74 out of 109 cases), and the expression of PGRMC1 was related to breast tumor size, lymph node metastasis, and prognoses. Patients with high PGRMC1 expression have lower disease-free survival as well as a lower overall survival, when compared with that of patients with low PGRMC1 expressions^74^. Even though there is limited information about PGRMC2, due to its 80% similarity to the cyt-b5 domain of PGRMC1, both of them might have overlapping functions and roles^72^. Causey et al.^75^ reported that PGRMC1 mRNA levels were significantly lower in stage II breast cancer patients than in stage III breast cancer patients, so measurement of PGRMC2 mRNA might be useful for the staging of breast adenocarcinoma.

As previously mentioned, clinical use of progestogens can be categorized into natural and synthetic progestins, both of which are derived from the pregnane skeleton (C21 backbone), with similarities as well as differences in their pharmacological properties^76^. The WHI EPT Trial^9^ reported that administration of CEE + MPA increased the risk of breast cancer in women, whereas a large observational study of E3N from France^12^ reported that administration of E2 + P4 or E2 + dydrogesterone did not increase the risk of breast cancer in women; these differences might be related to the pharmacological properties of different progestins.

### Natural progestogens

#### The P4 signaling pathways in breast cancer cells

P4 can stimulate the receptor activator of the NF-κB ligand (RANKL)^77–79^, receptor activator of NF-κB (RANK), PI3K/Akt^79–81^, and MAPK/ERK^82,83^ pathways by binding to the PR, which promotes the proliferation of breast cells. For example, with respect to the RANKL/RANK pathway, P4 promotes the proliferation of mammary stem cells^84^ and breast cells with the breast cancer 1 protein (BRCA1)^77^. The incidence of breast cancer in *BRCA1* mutation carriers is 2.4% and 1.7% in North America and Poland, respectively; these occurrences are significantly higher than the incidence of breast cancer in individuals without the *BRCA1* mutation^85^. Additionally, P4 induces cyclin D1 through the RANKL/RANK/IKK/NF-κB pathway^78^. Boopalan et al.^79^ reported that levels of cyclin D1 decreased in MCF-7 breast cancer cells when the PR was blocked by the selective progesterone receptor antagonist, mifepristone. With respect to the PI3K/Akt pathway, Wang et al.^80,81^ reported that P4 reduced the concentration of p27 in the nucleus and facilitated breast cancer cell proliferation through the PI3K/Akt pathway. In this process, P4 activated the PI3K/Akt pathway and subsequently increased the phosphorylation of p27 at T157 and pT198 sites, ultimately leading to the retention of p27 in the cytoplasm. Activation of kinase-interacting stathmin induced the phosphorylation of p27 at serine 10 (S10) within the nucleus, thereby promoting the transfer of p27 from the nucleus to the cytoplasm. The transfer of p27 attenuated the inhibition of cyclin-dependent kinase activity in the nucleus, which ultimately led to cell proliferation. With respect to the MAPK pathway, P4 interacts with the SH3 structural domain of the cytoplasmic signaling molecules through the amino-terminal polyproline motif of PR and activates the MAPK pathway, thereby affecting the transcription of cyclin D1 (CCND1)^82^. Additionally, Wang^86,87^ reported that P4 promoted the proliferation and migration of breast cancer cells associated with ERK activation resulting from the direct binding of PR and the SH3 domain of cellular Src (c-Src).

#### Effects of progesterone metabolites on breast cancer cells

**Figure 4:**
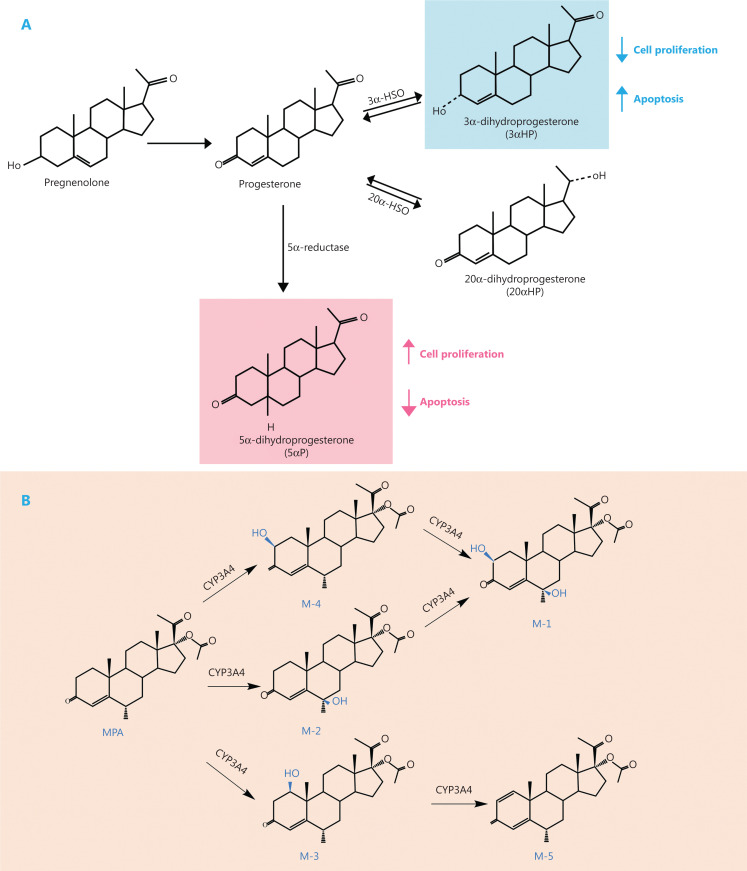
Metabolic pathways of progesterone (A) and medroxyprogesterone acetate (MPA) (B). A: Progesterone can be reduced to 5α-pregnane-3,20-dione (5αP) by 5α-reductase, or be converted to 3αHP by 3α-hydroxysteroid oxidoreductase (3α-HSO), or be metabolized to 20αHP through 20α-HSO in breast cells. The 5αP promotes breast cancer cell proliferation and inhibits breast cancer cell apoptosis, while 3αHP does the opposite. Abbreviations: 3α-HSO, 3α-hydroxysteroid oxidoreductase; 20α-HSO, 20α-hydroxysteroid oxidoreductase. B: the metabolic pathways of MPA in human liver microsomes.

Many tissues have progesterone-metabolizing enzymes, that act on different parts of the progesterone molecule.^88^ In breast cancer cells, P4 is converted into various products by different enzymes (**Figure 4A**). It can be reduced to 5α-dihydroprogesterone [also known as 5α-pregnane-3,20-dione (5αP)] by 5α-reductase, it can be converted to 3α-dihydroprogesterone [also known as 4-pregnen-3α-ol-20-one (3αHP)] by 3α-hydroxysteroid oxidoreductase (3α-HSO), or it can be metabolized to 20α-dihydroprogesterone [also known as 4-pregnen-20α-ol-3-one (20αHP)] by 20α-hydroxysteroid oxidoreductase (20α-HSO).

Previous studies^89,90^ reported that 5αP inhibited apoptosis and promoted the proliferation of breast cancer cells. In contrast, 3αHP promoted apoptosis and inhibited the proliferation of breast cancer cells. Due to certain technical limitations, it was difficult to obtain precise *in vivo* measurements of low progesterone levels in postmenopausal woman; hence, only a few studies have examined the probable effects of progesterone metabolites on breast cancer cells.

Trabert et al.^91^ improved the detection limit of progesterone from 3 ng/dL to 0.1 ng/dL using liquid chromatography-tandem mass spectrometry, which has significantly contributed to further studies of the association of progesterone metabolites with the risks of breast cancer. A previous study^89^ reported that the ratios of 5αP/3αHP in breast tumors and nontumor tissues of breast cancer patients were 18.16 ± 1.3 and 0.61 ± 0.16, respectively, thereby revealing a nearly 30-fold difference between the 2 groups. Moreover, Trabert et al.^17^ reported that the 5αP concentration (mean: 8.0; SD: 3.6) in the blood of 405 breast cancer patients was approximately 3-fold higher than the 3αHP concentration (mean: 2.5; SD: 1.3) in the B˜FIT cohort (as previously described). However, the B˜FIT cohort^17^ results reported that the ratio of 5αP/3αHP was not associated with an increased risk of postmenopausal breast cancer (HR: 1.00; 95% CI: 0.97–1.04; *P* = 0.85). Nevertheless, among women in the lowest tertile of 3αHP (< 1.72 ng/dL), those who belonged in the highest tertile of 5αP (> 9.12 ng/dL) were associated with an almost double risk of breast cancer (T3 *vs.* T1; HR: 1.96; 95% CI: 1.01–3.81; *P* = 0.04; *P* = 0.08 for interaction) when compared to the women in the lowest tertile of the 5αP group. Initially, these data did not support the hypothesis that 5αP exposure was associated with an increased risk of breast cancer. However, genetic variations in 5α-reductase activity in breast tissues might lead to differences in the 5αP concentrations in different individuals; hence, serum measurements might not be indicative of the real levels^92^. In the future, further studies are required to determine the exact roles 5αP and 3αHP in the development of breast cancer, as well as to verify whether they can be used as molecular markers for detecting the increased risk of breast cancer.

In summary, P4, which plays an essential role in normal breast development during puberty and pregnancy, might be a potent driver of breast cancer cell proliferation by multiple signaling pathways. Previous studies indicated that P4 metabolites, namely 5αP and 3αHP, had contrasting effects on breast cancer cell proliferation. These results contributed to a comprehensive understanding of the effects of P4 on breast cancer cell proliferation, which provide suggestions for further studies.

### Progestins

#### MPA

P4 is a C21-steroid hormone in which the pregnane skeleton contains 2 ketone groups (3, 20-dione), 1 each at the C3 and C20 positions, with a double bond between the C4 and C5 atoms^6^. MPA is a synthetic derivative of P4, and its progestogenic effect is similar to that of P4. However, even small structural changes might cause large variations in the functional effects of a molecule; for example, when compared with P4, MPA shows a relatively high progestogen activity when a methyl group is added to C6^93^.

Similar to P4, MPA can also promote mammary cell proliferation through the PR-mediated activation of RANKL/RANK, MAPK, PI3K/Akt, signal transducer, and activator of transcription 3 (Stat3), and other pathways. For example, with respect to the RANKL/RANK pathway, Schramek et al.^94^ reported that the MPA-induced proliferation of breast cancer cells was significantly reduced in RANK knockout Cre mice. With respect to the PI3K/Akt and MAPK/ERK pathways, Saitoh et al.^95^ reported that MPA induced CCND1 expression through the PI3K/Akt/NF-κB pathway. Similarly, Giulianelli et al.^96^ reported that MPA simultaneously recruited PR and ERα to the promoters of CCND1 and MYC, thereby promoting their expressions, and the MAPK/ERK, PI3K/Akt, and JAK/STAT pathways were involved in this process. With respect to the Stat pathway, Elizalde et al.^97,98^ reported MPA significantly upregulated P21^CIPI^ and CCND1 expressions in T47D breast cancer cells, resulting from assembling the Stat3/ErbB-2/PR transcriptional complex, where Stat3 bound to the P21^CIPI^ proximal promoter while both ErbB-2 and PR functioned as Stat3 co-activators. Furthermore, P21^CIPI^ and CCND1 were essential for MPA-driven breast cancer growth, both *in vitro* and *in vivo*.

Additionally, MPA activates the ERK and JNK pathways in MCF10A cells (PR-negative benign breast epithelial cells) through mPR, which could help us to better understand the importance of MPA and mPR in the development of breast cancer^99^.

However, unlike P4, MPA has a high affinity for the glucocorticoid receptor (GR)^100^. Courtin et al.^101,102^ reported that MPA facilitated the expression of fatty acid synthetase (FAS) through GR in MCF-7 breast cancer cells, and that FAS was closely related to the development of breast cancer. Furthermore, MPA displays a high affinity for androgen receptor (AR), and it can affect the proliferation of breast cancer cells through the AR signaling pathway^103^. It has been reported that administration of a high concentration of MPA (100 nM) could inhibit cell growth by activating the AR^103–105^, and a high concentration of MPA (> 500 mg/day) has been previously used as hormonal therapy for treating advanced breast cancer patients^106^. Birrell et al.^103^ reported that low concentrations of MPA (< 10 nM) exhibited an anti-androgen role, thereby interfering with the AR signaling pathway^107^. This interpretation was based on experiments in which MPA inhibited DHT-induced AR-N/C interactions^103^. However, this assumption is controversial because the inhibition of N/C interactions did not necessarily reflect the activity of an antagonist^108^.

#### Effects of MPA metabolites

**Figure 5:**
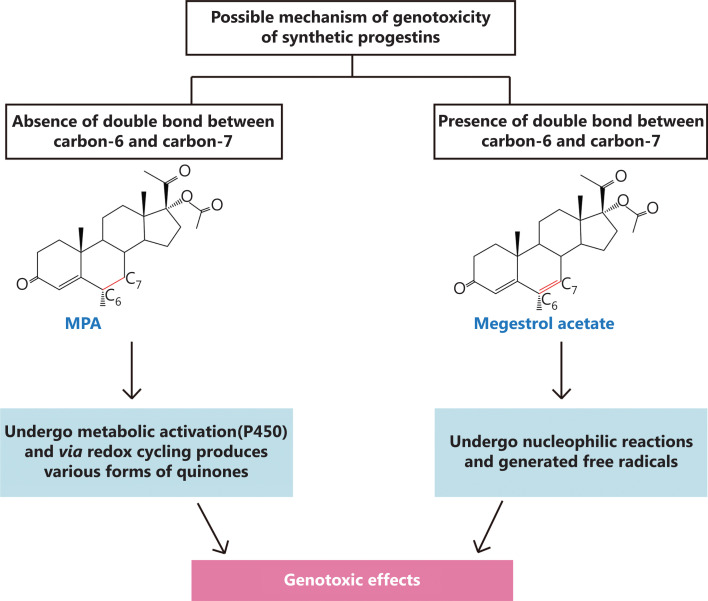
Possible mechanism of genotoxicity of synthetic progestins. The presence of the double bond between carbon 6 and carbon 7 might be significant for genotoxicity. Figure adapted from Siddique et al.^112^.

In the USA, MPA is the most commonly used progestin for MHT^109^. Siddique et al.^110^ suggested that MPA underwent metabolic activation to generate genotoxic ROS through cytochrome P450- and NADPH-dependent processes. Previously, a study on synthetic progestins reported that the double bond between carbon 6 (C6) and carbon 7 (C7) might be significant with respect to genotoxicity^110^. MPA lacks the double bond between C6 and C7, so it produces various forms of quinones through redox cycling, which can lead to genotoxic effects. In contrast, megestrol acetate might undergo nucleophilic reactions and generate free radicals due to the presence of the C6-C7 double bond^111^ (**Figure 5**). The CYP-mediated biotransformation usually serves as the first step in steroid elimination. CYP3A4 is the major cytochrome P450 isoform involved in the metabolism of MPA^113^. The formation rate and relative abundances of the MPA metabolites follows the order, M-2 > M-4 > M-3 > M-5 ≈ M-1 in human liver microsomes^113^ (**Figure 4B**). M-2, M-3, and M-4 are the dominant metabolites, accounting for more than 85% of the 5 metabolites. Although only MPA is considered as the active form^113^, the biological activities of its metabolites need to be further studied to determine their relationships with breast cancer. Moreover, the mechanism of MPA metabolism within the breast tissue is still not clear. Therefore, a comprehensive study of the metabolic pathways of MPA is needed.

Although many epidemiological studies have shown that the use of MPA in EPT was associated with an increased risk of breast cancer, the mechanism by which MPA affects the progression of breast cancer has not been fully elucidated. It is known that MPA binds to multiple steroid hormone receptors, such as PR, mPR, GR, and AR, and promotes the proliferation of breast cancer cells. However, administering higher and lower than normal doses of MPA has opposing biological effects on breast cancer proliferation due to their effects on the AR signaling pathway. Moreover, the mechanism by which MPA is metabolized in breast cancer cells and whether MPA metabolites increase the risk of breast cancer are yet to be determined.

#### NET

NET, which is mainly used in northern Europe, is a progestin compound with structural similarities to testosterone. In addition to its progestogenic effects, it also has very weak androgenic and estrogenic effects^109,114^. Previous studies^115,116^ have reported that 10^−8^ M–10^−5^ M NET significantly promoted the growth of MCF-7 cells overexpressing PGRMC1, and that this proliferative effect could be inhibited by administering the PGRMC1 antagonist, AG-205. Willibald et al.^117^ discovered that protein kinase CK2 (formerly known as casein kinase II) was involved in the phosphorylation of the PGRMC1 Ser181 site in MCF7/PGRMC1 cells (MCF-7 cells overexpressing PGRMC1) treated with NET, and that this was a prerequisite for the NET-induced proliferation of MCF7/PGRMC1 cells. Protein kinase CK2 is a serine/threonine protein kinase that is widely expressed and highly conserved in eukaryotic cells^118^. It is involved in various cellular processes, including metabolism, proliferation, differentiation, and apoptosis^119^. Moreover, previous studies^120,121^ reported that the E2/NET combination enhanced the proliferation of PGRMC1-overexpressing breast cancer cells, both *in vivo* and *in vitro*. The MWS observational study^10^ reported that the relative risk of using estrogen + NET with respect to the incidence of breast cancer was 1.53 (95% CI: 1.35–1.75), which was consistent with numerous previous studies. In contrast, in another study, the Danish Osteoporosis Prevention Study (DOPS; *n* = 1,006)^23^, reported that administration of E2 + NET did not increase the risk of breast cancer.

Because PGRMC1 is expressed in breast tissue and overexpressed in breast cancer tissue^73^, the molecular mechanism of PGRMC1 associated with the proliferation of breast cancer cells requires further study. It is important to determine whether breast cells overexpressing PGRMC1 are more likely to develop into tumor cells in women receiving E2/NET hormone therapy, which would help to determine the risks of MHT in relation to breast cancer.

## Conclusions and perspectives

By combining the results of recent MHT clinical trial studies, it has been suggested that there is a lower risk of developing breast cancer in women who use ET and E2 + P4, when compared to the breast cancer risk in women who use E2 + MPA. However, there are differences among the clinical trials with respect to the basic profile of the study population (age composition, time of menopause, and time of initiation of MHT), drug use (estrogen and progestin type, dosage, dosage form, and time of administration), dosing regimen (sequential or combined), and route of administration. Due to the great heterogeneity of these studies, it is difficult and inappropriate to reach a unified conclusion. Hence, long-term studies on the associations between increased risks of breast cancer and the underlying mechanisms of hormone therapy using different estrogen and progesterone regimens in early menopausal women are needed^108^.

To understand the molecular mechanisms of estrogen and progestogens and their relationships with breast cancer cell proliferation, we analyzed previous studies on estrogen and progesterone receptors in breast cancer cells as well as their signaling pathways, respectively. The genomic and non-genomic effects mediated by the hormone receptors played an important role in the proliferation of breast cancer cells. Furthermore, the biological functions of estrogen and progestogen metabolites were also linked with the proliferation of breast cancer cells, although this area of research required further study. Current studies on the metabolites of E2, MPA, and NET have focused on liver tissues. Notably, the metabolic pathways of all 3 were similar, and were associated with cytochrome P450 enzymes. Because E2 and MPA lack the C6-C7 double bond, they can generate various quinones through redox cycling, to produce genotoxic effects. However, the relevant metabolic enzymes and metabolic pathways of E2, MPA, and NET in breast tissue are still not clear, and the concentrations of their metabolites are difficult to measure by conventional methods due to their low concentrations. These limitations provide a challenge for further research dealing with the associations of the estrogen and progestogen metabolites with the risks of breast cancer.
